# The culinary medicine elective “Prospective Physicians For Fibre” improves fibre intake and nutrition knowledge in German medical students

**DOI:** 10.1186/s12909-025-08547-z

**Published:** 2026-01-06

**Authors:** Frieda Stübing, Julian Herter, Roman Huber, Megan F. Lee, Maximilian Andreas Storz

**Affiliations:** 1 Department of Internal Medicine II, Centre for Complementary Medicine, Medical Center – University of Freiburg, Faculty of Medicine, University of Freiburg, Freiburg, Germany; 2 Bond University, Faculty of Society and Design, Gold Coast, Australia

**Keywords:** Culinary medicine, Nutrition, Dietetics, Medical education, Dietary fibre, Nutrient intake

## Abstract

**Background:**

Nutrition education remains an underdeveloped component of medical training globally. This gap in knowledge can hinder physicians from effectively integrating nutrition into clinical practice, creating a significant barrier to supporting meaningful dietary changes in patients. We developed a culinary medicine elective for 3rd to 6th year medical students titled “Prospective Physicians For Fibre (PPFF)” to promote students’ knowledge about the importance of fibre and its benefits in health and disease.

**Methods:**

The four-week fibre-focused, mixed-methods, short-term, culinary medicine elective PPFF was tested in a non-controlled pilot study. Students’ fibre intake and knowledge about fibre were compared in a pre-post design in *n* = 47 participants.

**Results:**

Participants had a median age of 24 years; females accounted for 72% of the sample. Mean fibre intake based on 2-day weighed food diaries increased from 29.52 ± 10.72 to 38.40 ± 12.14 g/d after the elective (*P* < .001). Prior to the elective, self-assessed fibre intake ranged from 5 to 1000 g/d, and was not correlated with food diary-based fibre intake. After the elective, self-assessed fibre intakes were more accurate and a strong correlation between self-assessed and actual fibre intake was observed (Spearman’s rho = 0.55, *P* < .001). Students’ scoring in the PPFF survey improved significantly from 3.06 ± 1.66 to 7.70 ± 1.49 points (*P* < .001). The elective received very good ratings and increased students’ self-perceived competences in various domains, such as fibre intake assessment, nutrition counselling and cooking skills.

**Conclusions:**

PPFF improves fibre intake and nutrition knowledge in a pilot cohort of German medical students and may equip students with essential skills and competencies for their future career.

**Supplementary Information:**

The online version contains supplementary material available at 10.1186/s12909-025-08547-z.

## Background

Dietary and lifestyle modifications are a first-line intervention in the treatment of many diseases [1]. Nevertheless, surveys with physicians frequently suggest a shortfall in diet counselling competencies [1, 2]. The gap between dietary fibre intake recommendations and actual fibre consumption is large in many Western countries, having substantial implications for public health [3, 4]. Physicians are regarded as trusted communicators, and the medical community increasingly acknowledges the crucial role of nutrition in health and disease [5]. A lack of nutrition training and education in health professionals, such as physicians, however, is also well documented and subject to intensive debates [6, 7].

The shortage of knowledge, practical skills and the lack of compensation for nutrition counselling impede physicians from translating nutrition priorities into practice, and poses an important barrier to detailed and sustained nutrition advice that results in meaningful dietary changes for patients [5, 8].

There is now a consensus that medical students as prospective physicians *must* be better equipped with nutritional competencies [9]. Yet, the state of nutrition education in medicine is inadequate, with nutrition-related topics being poorly integrated into lectures and examinations [10, 11]. Most medical students receive only a few contact hours of nutrition instruction during their entire time at medical school [6, 12]. As a consequence, prospective physicians neither feel sufficiently equipped with knowledge nor confident enough to give nutritional recommendations to patients [9]. Innovative solutions to this dilemma are urgently required.

Culinary medicine is a field of science that blends food and cooking with the science of medicine [13]. It focuses on educating individuals about the interconnection between dietary choices and chronic disease through practical, hands-on approaches to nutrition [12]. Culinary medicine electives have been suggested as an effective means to tackle the problem faced by graduating medical students with a lack in nutrition education [1, 13]. Culinary medicine has substantially increased in popularity in the United States of America over the last decades [14], and an expert panel recently recommended that nutrition competencies should be assessed as part of licensing exams in the United States of American [9]. However, such approaches are scarce in European medical education, and with the exception of Göttingen, Giessen, and Brandenburg, they are *– to the best of our knowledge –* non-existent in medical school curricula in Germany [15].

We addressed this gap and developed an evidence-based, fibre-focused, mixed-methods, short-term, culinary medicine elective for clinical medical students titled “Prospective Physicians For Fibre (PPFF)”. This four-week elective was designed to promote students’ knowledge about the importance of fibre and its benefits in health and disease. In this study, we implemented and tested the elective in the context of a non-controlled pilot study and hypothesised that participation in the elective would improve students’ knowledge about fibre and increase their dietary fibre intake, in turn, preparing them more sufficiently for giving nutrition advice in their fields upon graduation.

## Methods

### Study design

**Fig. 1:**
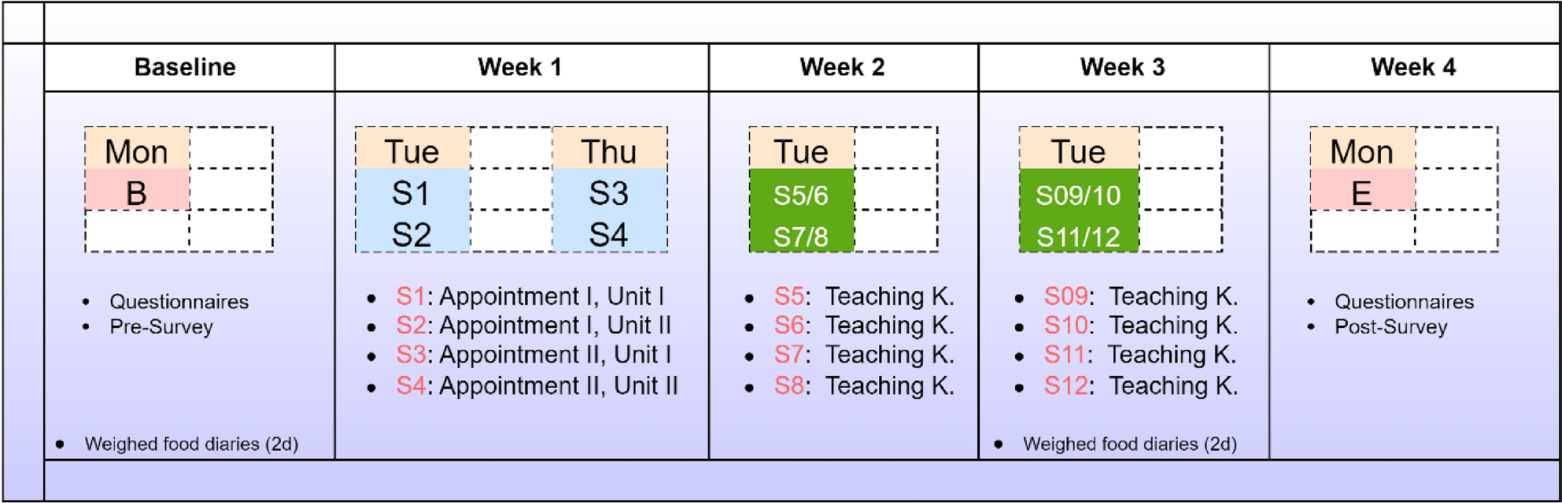
Course of the study: an overview. Mon = Monday; Tue = Tuesday; Thu = Thursday; B = Baseline; E = End; Teaching K. = Teaching Kitchen; S = Session. Session details are also discussed in Supplementary Table 1. Each session had a duration of 60 minutes

This monocentric and non-controlled pilot study was conducted between April and June 2025. Data were analysed in July and August 2025. Figure 1 displays the course of the study. Participants were recruited via advertisements on notice boards at Freiburg University and via social media (Meta Platforms). Potentially eligible study participants who signalled interest in the study were initially screened by phone. Those who met the inclusion criteria were subsequently invited for a baseline screening (Fig. 1). The purpose of the study was explained, and participants were then asked to complete the study-specific survey. Prior to the first study session, we asked all participants to complete a two-day weighed food diary, for which participants received detailed instructions. The first study week included four sessions outside the teaching kitchen (50 min each) in total. The second and third study weeks each included four sessions in the teaching kitchen.

Afterwards, participants were asked to complete another round of weighed food diaries. Finally, we invited participants for a concluding face-to-face conversation and the post survey. The purpose of the concluding conversation was to discuss students’ positive and negative experiences with the elective and to collect input for future elective rounds. Participants who attended all sessions and who handed in four complete, weighed food diaries received a financial remuneration of 100€. Study participants were divided into four groups with a maximum size of *n* = 12 participants each. The rationale for limiting the group size was to allow for an intensified teaching experience in small groups and to maximise practical hands-on exercise.

### Inclusion and exclusion criteria

Only medical students at Freiburg University Medical Center aged 18 to 60 years were included in this study. All students had to provide a current certificate of enrolment upon entering the study. The German medical curriculum and training is divided into three sections: (I) a first basic science part (2 years), (II) a clinical science part (3 years) and (III) a final clinical year [16]. Only students who successfully passed the first basic science part were eligible for this study.

Exclusion criteria focused on excluding participants with expected difficulties with an increased fibre intake. Individuals with known gastrointestinal tract diseases (e.g., celiac disease or inflammatory bowel syndrome), were excluded, as a sudden increase in dietary fibre intake could be problematic and create gastrointestinal distress. Additional exclusion criteria included breastfeeding or pregnant women, those with self-reported eating disorders or obesity (body mass index > 30 kg/m²) - which may require a special diet (e.g., a low-carbohydrate diet) or caloric restriction, regular alcohol intake (> 20 g/d), daily smokers, and those with psychiatric illness not compatible with group intervention.

### Sessions outside the teaching kitchen

Sessions 1–4 were conducted outside the teaching kitchen (Fig. 1). Each session had a duration of 50 min. The topic and content of each session may be obtained from Supplementary Table 1. In brief, we began with an introduction session emphasising the goals and purpose of the culinary medicine elective. This session also included instructions on how to complete the food diaries. It also included a brief self-introduction of each participant (e.g., motivation for undertaking the elective, number of clinical semesters, age at participation, etc.). Session 2 was focused on nutrient intake assessments with a particular focus on dietary fibre. This session covered topics such as daily fibre requirements, physiological properties of fibre, as well as practical food-based exercises in which students determined the fibre content of various foods in small groups with *n* = 4 students. Results were then discussed in the larger group. Real foods were provided. Session 2 also focused on flour, fibre processing, and fibre intake-related challenges with gluten-free foods.

Session 3 focused on clinical case files, in which the previously discussed knowledge was put into practice in case-based discussions. Three cases were interactively discussed (colorectal cancer, metabolic syndrome, and constipation). Session 4 covered the role of legumes in ensuring adequate fibre intake. Nutritional properties of legumes and the role of legume processing were discussed. This session included hands-on experiences with the different legumes provided. The focus was put on legume identification, legume preparation and nutritional properties of dried and canned legumes.

### Sessions in the teaching kitchen

Sessions 5–12 were conducted in the teaching kitchen. As Freiburg University has no own teaching kitchen, we rented a professional kitchen for the respective sessions. The kitchen was fully equipped and can be rented on a per-hour basis for cooking sessions with larger groups. Each session had a duration of 60 min of hands-on experience. Sessions included basic culinary skills including essential kitchen safety and cooking techniques. Sessions took place in the evening starting at 6 pm. Students prepared various meals under the supervision of a professional chef who led all sessions and who got paid for this service. All meals were plant-based and vegan for practical purposes (e.g., with regard to food allergies) and to increase fibre intake. Two different three-course meals rich in dietary fibre were prepared based on the chef’s recommendations (Supplementary Table 2). Afterwards, participants ate together at the teaching kitchen. Beverages including tap and sparkling water, tea, coffee and juice were provided by the teaching kitchen. Faculty was present at the majority of sessions (with the exception of one evening which was missed due to sickness) and available for questions. Before leaving the kitchen, students were asked to help the available stuff with the clean-up.

### The pre- and post-survey

Participants were asked to complete a knowledge-based survey prior to entering the study and after completing the study. The survey included 10 multiple-choice questions which all covered topics related to dietary fibre and legumes as discussed in study sessions. Some survey questions had only one correct answer, whereas others were multi-select questions. The questions are shown in Supplementary Table 3. The number of correct answers was compared pre-post.

### Nutrient intake assessment

Participants were asked to complete weighed food diaries on two occasions during the study (four days in total). Nutrient intake was calculated based on the average of two days. The employed methods have been discussed in great detail in previous publications [17–19]. In brief, we asked all participants to quantify their consumed foods and beverages to the nearest 0.1 g or ml. Participants were provided with calibrated precision scales (Wedo Elektronische Universalwaage Optimo5000, Dieburg, Germany or a comparable model) and detailed instructions on how to complete the food diaries. When exact weighing was impossible (e.g., when eating at the cafeteria), participants were allowed to provide semi-quantitative household recordings with common measures (spoons, cups, etc.) [20]. The protocols were analysed using NutriGuide^®^ plus software (Version 4.9, Nutri-Science GmbH, Hausach, Germany).

### Primary and secondary outcome(s)

The primary outcome was the change in daily fibre intake in g/d before and after completion of the PPFF elective. Secondary outcomes included (I) performance in the survey (percentage of correct answers) before and after completion of the elective; (II) discrepancy and correlation between self-assessed fibre intake and fibre intake as determined by the completed two-day weighed food diaries (before and after completion of the PPFF elective); and (III) a descriptive assessment of the drop-out rate (e.g., how many participants withdrew from the study, at what stage of the study, and why?).

### Statistical analysis

Based on the available literature and our experience with nutrition studies, a small effect size for changes in fibre intake was assumed. The corresponding sample size estimation was performed in Statulator using the “sample size calculator for paired differences comparison” module [21]. According to Statulator, the study would require a sample size of *n* = 43 pairs to achieve a power of 80% and a level of significance of 5% (two-sided) for detecting an effect size of 0.44 between pairs. We assumed a drop-out rate of approximately 10% and thus aimed to recruit *n* = 48 participants. The statistical analysis was performed in Stata 18 (StataCorp, 2023. Stata Statistical Software: Release 18. College Station, TX: StataCorp LLC).

Box plots and the Shapiro-Wilk test were used to test for normality. Normally distributed variables were described with their mean and standard deviation, whereas categorical variables were described as the number of observations (percentage). Variables that were not normally distributed were described with the median and interquartile range in parentheses. Strip plots, Bland-Altmann difference plots and scatterplots were used to visualise the data. Pearson’s product-moment correlations and Spearman’s rank-order correlations were run to assess the relationship between self-estimated and actual dietary fibre intake. The Wilcoxon signed-rank test and the paired t-test were used to assess differences in fibre intake and in the overall score in the study-specific survey prior to and after the PPFF elective. The Stuart-Maxwell Test was used to compare the marginal distributions of paired categorical variables. Crude fibre intake and fibre intake/1000 kcals were compared to account for the inhomogeneous study population. Alpha was set to 0.05. The reporting of this study followed and complies with the DoCTRINE Guidelines: Defined Criteria To Report INnovations in Education [22].

## Results

The final study population included *n* = 47 participants (see Supplementary Fig. 1). All participants who entered the study completed it successfully. Table 1 displays the sample’s characteristics. Participants had a median age of 24 years; females accounted for 72% of the study population. The study attracted students from all clinical semesters, and almost 43% indicated some (but limited) prior nutrition education.

**Table 1:** Sample characteristics (based on *n* = 47 observations)

Variable	
***Sex***	
Male	*n* = 13 (27.66%)
Female	*n* = 34 (72.34%)
***Highest education level***	
German “Abitur”	*n* = 46 (97.87%)
University degree	*n* = 1 (2.13%)
***Origin*** ^***a***^	
Germany	*n* = 40 (86.96%)
France	*n* = 5 (10.87%)
Bulgaria	*n* = 1 (2.17%)
***Marital status***	
Single	*n* = 24 (51.06%)
With partner, not married	*n* = 23 (48.94%)
***Diet***	
Omnivorous diet	*n* = 32 (68.09%)
Vegetarian diet	*n* = 12 (25.53%)
Vegan diet	*n* = 3 (6.38%)
***Current semester***	
5	*n* = 2 (4.26%)
6	*n* = 10 (21.28%)
7	*n* = 2 (4.26%)
8	*n* = 6 (12.77%)
9	*n* = 6 (12.77%)
10	*n* = 11 (23.40%)
> 10	*n* = 10 (21.28%)
***Prior nutrition classes***	
Yes	*n* = 20 (42.55%)
No	*n* = 27 (57.45%)
***Median hours of nutrition classes*** ^***b***^	2 (2.25)
*Age (years)*	24 (4)
*Weight (kg)*	64 (23.5)
*Height (cm)*	172.83 ± 8.41
*BMI (kg/m*^*2*^*)*	22.04 (4.02)
*MacArthur Scale of Subjective Social Status (range: 0–10)*	6.70 ± 1.10
***Desired speciality***	
Internal medicine	*n* = 8 (17.02%)
Pediatrics	*n* = 2 (4.26%)
General medicine	*n* = 3 (6.38%)
Surgery	*n* = 3 (6.38%)
Anesthesiology	*n* = 1 (2.13%)
Psychiatry	*n* = 1 (2.13%)
Dermatology	*n* = 2 (4.26%)
Gynecology/Obstetrics	*n* = 5 (10.64%)
Orthopedics	*n* = 2 (4.26%)
Opthalmology	*n* = 1 (2.13%)
Undecided	*n* = 16 (34.04%)
Others	*n* = 3 (6.38%)

Continuous data displayed as mean ± SD if normally distributed or as median (IQR) if not normally distributed. Categorical data displayed as *n* = x (%). ^a^ based on *n* = 46 observations only as one person did not disclose her origin. ^b^ based on *n* = 20 observations indicating prior nutrition classes

Table 2 suggested a poor knowledge about fibre and its role in health and disease in the sample *prior* to the elective. On average, 3.06 fibre-related multiple-choice questions were answered correctly. Self-estimated fibre intake per day ranged from 5 to 1000 g/d, with a median of 50 (170) g/d.

**Table 2:** Survey results and fibre intakes: pre vs. post comparison (based on *n* = 47 paired observations)

Outcomes	Prior to the elective	Post elective	*P*-value
***Primary outcome:***			
Food diary-based fibre intake (g/d)	29.52 ± 10.72	38.40 ± 12.14	**< 0.001**
Food diary-based fibre intake (g/1000 kcal)	13.28 ± 4.71	17.20 ± 4.84	**< 0.001**
***Secondary outcomes***:			
PPFF survey score (range: 0–10 points)	3.06 ± 1.66	7.70 ± 1.49	**< 0.001**
Self-assessed fibre intake (g/d)	50 (170)	25 (12.5)	**< 0.001**
Median difference between self-assessed and food diary-based fibre intake (g/d)	27.96 (163.79)	12.53 (8.68)	**< 0.001**
Median difference between self-assessed and food diary-based fibre intake (g/1000 kcal)	41.18 (171.82)	8.97 (11.06)	**< 0.001**

Continuous data displayed as mean ± SD if normally distributed or as median (IQR) if not normally distributed. Based on absolute differences

**Fig. 2:**
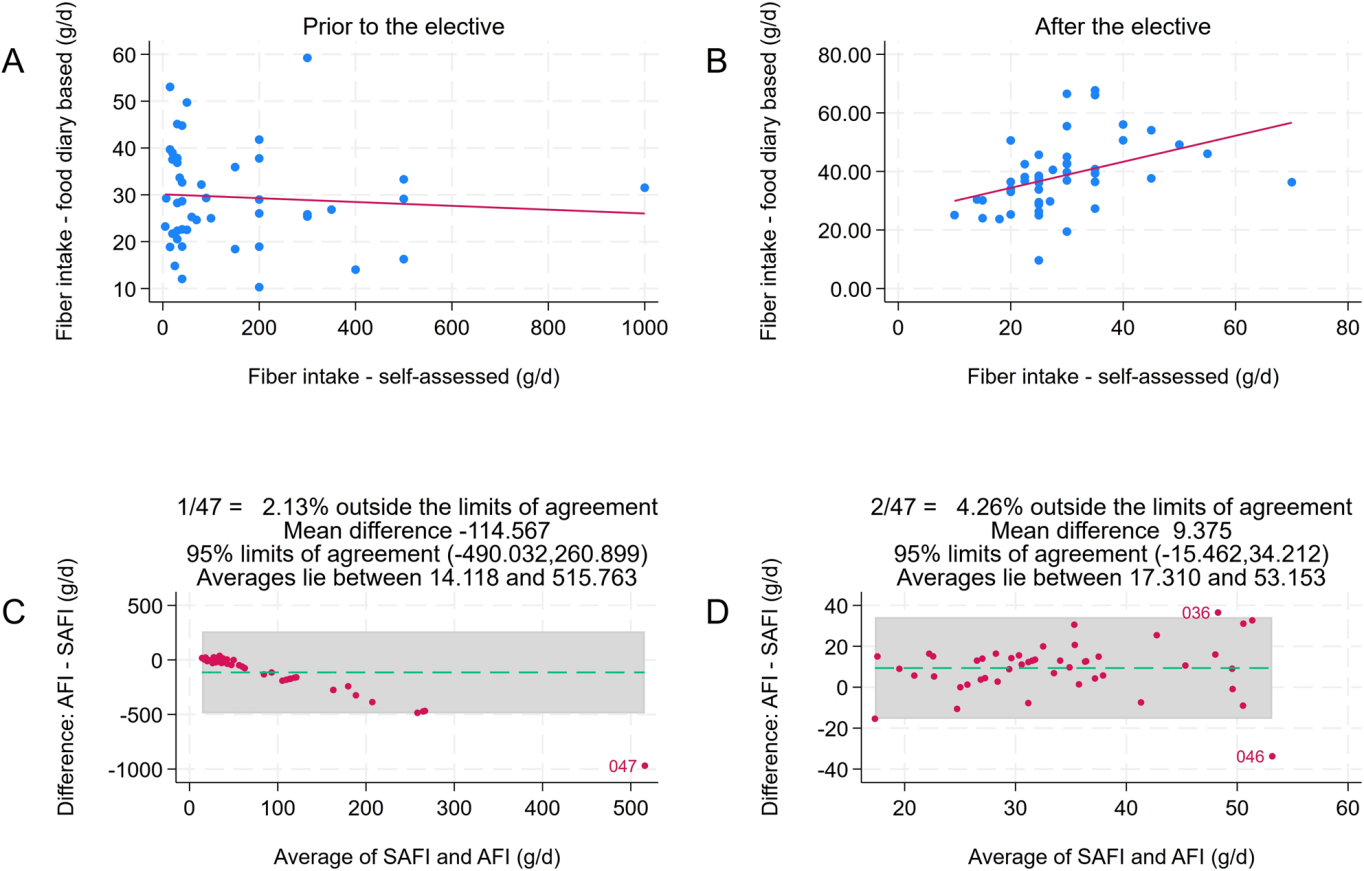
Correlation analysis: estimated *vs*. actual weighed food diary-based fibre intake. Panel (**A**) shows the correlation prior to the elective, whereas panel (**B**) displays the correlation after completion of the elective. After the elective, a strong correlation between self-assed fibre intake (SAFI) and actual fibre intake (AFI) was found (Spearman's rho = 0.55, *P*<.001). The Bland-Altman difference plots in panels (**C**) and (**D**) illustrate the better alignment of actual *vs.* estimated fibre intake after the elective

Figure 2 displays a scatterplot in which estimated *vs*. actual weighed food diary-based fibre intake was correlated *prior* to the elective. The non-significant correlation between both variables suggested a poor knowledge about fibre *before* the elective. After completing the elective, the participants scored significantly better in the study-specific survey. On average, 7.70 questions were answered correctly. The number of correctly answered questions increased on average by 4.63 (95%-confidence interval: 4.16- 5.12).

The alignment between self-estimated and actual fibre intakes based on the weighed food diaries was much better after the elective, as indicated by the higher correlation coefficient and the Bland-Altman difference plot in Fig. 2. Absolute fibre intake in participants increased on average by 8.88 g/d (95%- confidence interval: 6.29–11.47 g/d).

Participation in the PPFF elective resulted in short-term intake improvements for several nutrients. Table 3 displays a pre-post comparison for 13 macro- and micronutrients. The fact that participants consumed more plant foods to increase their fibre intake also resulted in a higher intake of magnesium and a lower intake of saturated fatty acids.

**Table 3:** Nutrient intake data: pre vs. post comparison (based on *n* = 47 paired observations)

Nutrient	Prior to the elective	Post elective	*P*-value
Energy (kcal/d)	2112 (645.50)	2174 (808)	0.958
Energy (kJ/d)	8836.61 (2700.77)	9096.02 (3380.67)	0.958
Calcium (mg/d)	1112.71 (642.04)	1134.22 (563.83)	0.280
Carbohydrates (g/d)	220.41 (73.49)	243.69 ± 62.19	0.295
Fat (g/d)	97.20 (52.34)	92.69 (52.16)	0.258
Iron (mg/d)	15.72 ± 5.53	18.40 ± 6.55	**0.006**
Magnesium (mg/d)	420.67 (164.41)	487.59 (200.45)	**0.001**
Monounsaturated fatty acids (g/d)	33.82 (19.99)	33.71 (24.13)	0.849
Phosphorus (mg/d)	1902.05 (850.14)	1803.44 (1087.93)	0.941
Polyunsaturated fatty acids (g/d)	15.18 (12.44)	16.48 (12.92)	0.983
Potassium (mg/d)	3046.75 ± 834.75	3189.79 ± 859.99	0.251
Protein (g/d)	81.11 (35.12)	77.36 (39.56)	0.882
Saturated fatty acids (g/d)	32.44 (22.07)	27.89 (19.46)	**0.043**
Sodium (mg/d)	2613.60 (1756.31)	2771.53 (2172.29)	0.949
Zinc (mg/d)	10.38 (5.01)	10.24 (5.57)	0.064

Continuous data displayed as mean ± SD if normally distributed or as median (IQR) if not normally distributed

Overall, the attrition rate was surprisingly low. All participants who completed the baseline screening also completed the study. While participants received a remuneration of 100€ for completing the elective, responses in the course evaluation suggested other reasons explaining the low attrition rate. Most participants rated the elective with grade 1 (very good), the best grade available in Germany. Further to that, 95.74% strongly agreed that they would recommend the elective to their peers (Supplementary Fig. 2). Almost 94% agreed or strongly agreed that they would retake the elective even without remuneration.

Selected elective evaluations are summarised in Supplementary Table 4. Participating students rated the high proportion of hands-on experience and the practical food-based exercises as particularly positive. Participating students suggested expanding the elective and including other nutrients (including protein) as well as sessions on dietary supplements.

Finally, we constructed bar charts for the Likert scale-based variables in the study (Supplementary Figs. 3, 4, 5, 6 and 7). Pre-post comparisons suggested significant differences in students’ confidence in estimating the fibre content of foods (*P* <.001; Supplementary Fig. 3) and in students’ attention to the fibre content of foods when shopping (*P* <.001; Supplementary Fig. 4). After completing the elective, students indicated a significant difference in their ability to prepare pulses (Supplementary Fig. 4). Following the elective, students felt better equipped to talk with patients about food and health (*P* <.001; Supplementary Fig. 6). After the elective, most participants believed that every patient should receive focused nutritional history and dietary advice (*P* =.012). Supplementary Fig.** 7** displays pre vs. post-elective comparisons regarding students’ confidence in nutrition counselling and cooking skills, both improved significantly after the elective.

## Discussion

Dietary and lifestyle modifications are considered a first-line intervention in the treatment of many diseases [1]. Nevertheless, surveys with physicians frequently suggest a shortfall in diet counselling competencies [1, 2]. Hands-on culinary medicine electives have been identified as a promising tool to increase nutritional knowledge and confidence in dietary counselling in medical students [1].

In Germany, culinary medicine electives are scarce and rarely available, with only a few exceptions [15]. Results from the culinary medicine elective at the German medical schools of the universities of Göttingen, Giessen, and Brandenburg, published in 2023, suggest a significant increase in counselling competencies on 25 nutrition and lifestyle medicine topics [15]. Comparable electives in Germany are – *to the best of our knowledge* – non-existent.

For this reason, we developed “Prospective Physicians For Fibre (PPFF)”, a short-term evidence-based, culinary medicine elective for clinical medical students which focuses on fibre and its role in health and disease. The study was conducted as a pilot study to investigate acceptance and interest among students in Freiburg, Germany. All participants who passed the baseline screening finished the study, resulting in an attrition rate of 0%. Participants evaluated the elective very favourably, and the feedback towards its innovative design and its piloting character in Freiburg medical education was overwhelmingly positive. A reservation must be made; however, that participants also received a financial remuneration for completing the study. The major rationale was to compensate participants for the time-consuming task of completing food diaries.

Weighed food diaries were implemented because very few culinary medicine electives have investigated nutrient intakes using this particular method. Most electives focused on qualitative outcome measures, while quantitative outcome measures were often restricted to post-experience surveys with Likert-type rating scales [23], pre-post course surveys [24], and assessments of personal dietary/food choices [25]. Weighed food diaries are considered the gold standard for accurately measuring dietary intake, as they involve weighing all foods consumed [26]. Our results revealed a significant increase in fibre intake subsequent to the elective. These findings are particularly noteworthy, as culinary electives may primarily attract the interest of students with a pre-existing interest in nutrition (and thus a potentially better nutrient intake).

Prior to the elective, a high percentage of participants did not meet the national intake recommendations for fibre (30 g/day). This changed upon completing the elective, with 74.47% reaching or surpassing the national intake recommendations. Notably, nutrient intakes for other nutrients of public health concern (magnesium) also increased, which overall suggests a better diet quality. We acknowledge that no long-term data were collected. Long-term improvements in diet quality are not to be expected after a short-term elective of just four weeks. Then again, a longer duration was not deemed feasible, given the pilot nature of this intervention.

Prior to the elective, most medical students were unable to answer basic fibre-related multiple-choice questions successfully. Approximately 53% of students could not correctly specify the national dietary fibre intake recommendation. Only a handful of students satisfactorily answered the multiple-choice question regarding dietary fibre sources. These findings align with a study by Howe et al. suggesting that healthcare providers’ lifestyle habits matter when it comes to lifestyle and nutritional counselling for patients [27].

The present study is not without limitations. The short elective duration, the modest number of participants and the lack of long-term data must be transparently acknowledged. Further to that, we acknowledge the possibility of a participant selection bias: Those who participated in the study may have been more interested in the subject matter which could have influenced the study outcomes. Strengths include the innovative design, the fibre-focused curriculum, the usage of quantitative outcomes using gold standard methodology (weighed food diaries) and the high share of interactive hands-on experience during the elective. Future studies may include additional nutrients and content, and should extend over a longer period than four weeks. This applies for both knowledge retention and long-term use of the acquired knowledge in clinical practice. For future elective rounds, we also seek to highlight the importance of interdisciplinary collaboration with dieticians.

Although dietary change can significantly improve patients’ health, clinicians rarely discuss nutrition with their patients [28]. Kahan and Manson; Crowley et al., and Mehrtash and Manson all discuss potential strategies to enhance medical education and primary care concerning nutrition [28–30]. Cumulative findings suggest that institutional commitment to make nutrition education a priority in medical training is key to providing students with high-quality nutritional education [28]. Crowley et al. emphasise that funding for innovative curriculum initiatives may substantially enhance medical nutrition education [29]. In this context, we would like to emphasise that the present study was funded by a grant of the University of Freiburg for innovative teaching projects (Studierendenvorschlagsbudget 2025). Grant money allocation and project selection for funding was performed by students, which clearly shows how much students desired nutrition education in their curriculum. We believe that hands-on experience in the context of a culinary medicine elective may achieve this goal and thus initiated this first study to potentially improve nutrition education in Freiburg, Germany.

## Conclusions

Institutional commitment to make nutrition education a priority in medical training is nowadays key to providing students with high-quality nutritional education. Culinary medicine electives with a high share of hands-on experience may help to reach that goal. Our study adds to the existing body of evidence surrounding culinary medicine and shows *- for the very first time with reference to the German context -*that such electives may have profound effects beyond self-rated gains in knowledge. Fibre intake, a nutrient of public health concern and closely linked to many non-communicable diseases, substantially increased in study participants upon completion of this elective.

## Supplementary Information

Supplementary Material 1.

## Data Availability

The underlying data is available from the corresponding author (MAS) on reasonable request.

## Abbreviations

PPFF: Prospective Physicians For Fibre

## References

[1] Tan J, Atamanchuk L, Rao T, Sato K, Crowley J, Ball L. Exploring culinary medicine as a promising method of nutritional education in medical school: a scoping review. BMC Med Educ. 2022;22(1):441. 35672843 10.1186/s12909-022-03449-w PMC9175378

[2] Kolasa KM, Rickett K. Barriers to providing nutrition counseling cited by physicians. Nutr Clin Pract. 2010;25(5):502–9. 20962310 10.1177/0884533610380057

[3] Thompson HJ, Brick MA, Perspective. Closing the dietary fibre gap: an ancient solution for a 21st century problem. Adv Nutr. 2016;7(4):623–6. 27422499 10.3945/an.115.009696 PMC4942856

[4] McKeown NM, Fahey GC, Slavin J, van der Kamp JW. Fibre intake for optimal health: how can healthcare professionals support people to reach dietary recommendations? BMJ. 2022;378:e054370. 35858693 10.1136/bmj-2020-054370 PMC9298262

[5] Storz MA. Is there a lack of support for whole-food, plant-based diets in the medical community? Perm J. 2018;23:18–068. 10.7812/TPP/18-068 PMC6307547 30589405

[6] Magallanes E, Sen A, Siler M, Albin J. Nutrition from the kitchen: culinary medicine impacts students’ counseling confidence. BMC Med Educ. 2021;21(1):88. 33541352 10.1186/s12909-021-02512-2 PMC7863372

[7] Adamski M, Gibson S, Leech M, Truby H. Are doctors nutritionists? What is the role of doctors in providing nutrition advice? Nutr Bull. 2018;43(2):147–52.

[8] Storz MA. Barriers to the plant-based movement: a physician’s perspective. IJDRP. 2020;2:4. 10.22230/ijdrp.2020v2n2a157.

[9] Eisenberg DM, Cole A, Maile EJ, Salt M, Armstrong E, Broad Leib E, et al. Proposed nutrition competencies for medical students and physician trainees: a consensus statement. JAMA Netw Open. 2024;7(9):e2435425. 39348126 10.1001/jamanetworkopen.2024.35425

[10] Storz MA, Oksche A, Schlasius-Ratter U, Schillings V, Beckschulte K, Huber R. Nutrition coverage in medical licensing examinations in germany: an analysis of six nationwide exams. Nutrients. 2022;14(24):5333. 36558492 10.3390/nu14245333 PMC9780865

[11] Mogre V, Stevens FCJ, Aryee PA, Amalba A, Scherpbier AJJA. Why nutrition education is inadequate in the medical curriculum: a qualitative study of students’ perspectives on barriers and strategies. BMC Med Educ. 2018;18(1):26. 29433505 10.1186/s12909-018-1130-5 PMC5809975

[12] Adams KM, Kohlmeier M, Zeisel SH. Nutrition education in U.S. medical schools: latest update of a National survey. Acad Med. 2010;85(9):1537–42. 20736683 10.1097/ACM.0b013e3181eab71b PMC4042309

[13] La Puma J. What is culinary medicine and what does it do? Popul Health Manag. 2016;19(1):1–3. 26035069 10.1089/pop.2015.0003 PMC4739343

[14] Newman C, Yan J, Messiah SE, Albin J. Culinary medicine as innovative nutrition education for medical students: a scoping review. Acad Med. 2023;98(2):274–86. 35921151 10.1097/ACM.0000000000004895

[15] Böttcher S, Schonebeck LJ, Drösch L, Plogmann AM, Leineweber CG, Puderbach S, et al. Comparison of effectiveness regarding a culinary medicine elective for medical students in Germany delivered virtually versus In-Person. Nutrients. 2023;15(19):4281. 37836565 10.3390/nu15194281 PMC10574049

[16] Chenot JF. Undergraduate medical education in Germany. Ger Med Sci. 2009;7:Doc02. 19675742 10.3205/000061 PMC2716556

[17] Storz MA, Müller A, Niederreiter L, Zimmermann-Klemd AM, Suarez-Alvarez M, Kowarschik S,et al. A cross-sectional study of nutritional status in healthy, young, physically-active German omnivores, vegetarians and vegans reveals adequate vitamin B12 status in supplemented vegans. Ann Med. 55(2):2269969. 10.1080/07853890.2023.2269969 PMC10586079 37851870

[18] Correction. Ann Med. 56:2346423. 10.1080/07853890.2024.2346423 PMC11073407 38701001

[19] Herter J, Stübing F, Lüth V, Zimmermann J, Lederer AK, Hannibal L, et al. Bowel health, defecation patterns and nutrient intake following adoption of a vegan diet: a randomized-controlled trial. Ann Med. 56(1):2305693. 10.1080/07853890.2024.2305693 PMC10854443 38327148

[20] Alexy U, Fischer M, Weder S, Längler A, Michalsen A, Sputtek A, et al. Nutrient intake and status of German children and adolescents consuming Vegetarian, vegan or omnivore diets: results of the VeChi youth study. Nutrients. 2021;13(5):1707. 34069944 10.3390/nu13051707 PMC8157583

[21] Dhand NK, Khatkar MS, Statulator. An online statistical calculator. Sample size calculator for comparing two paired means. 2014. http://statulator.com/SampleSize/ss2PM.html. Accessed 13 Nov 2025.

[22] Blanco M, Prunuske J, DiCorcia M, Learman LA, Mutcheson B, Huang GC. The doctrine guidelines: defined criteria to report innovations in education. Acad Med. 2022;97(5):689–95. 35171122 10.1097/ACM.0000000000004634

[23] Hashimi H, Boggs K, Harada CN. Cooking demonstrations to teach nutrition counseling and social determinants of health. Educ Health (Abingdon). 2020;33(2):74–8. 33318459 10.4103/efh.EfH_234_19

[24] D’Adamo CR, Workman K, Barnabic C, Retener N, Siaton B, Piedrahita G, et al. Culinary medicine training in core medical school curriculum improved medical student nutrition knowledge and confidence in providing nutrition counseling. Am J Lifestyle Med. 2022;16(6):740–52. 36389046 10.1177/15598276211021749 PMC9644147

[25] Rothman JM, Bilici N, Mergler B, Schumacher R, Mataraza-Desmond T, Booth M, et al. A culinary medicine elective for clinically experienced medical students: a pilot study. J Altern Complement Med. 2020;26(7):636–44. 32543207 10.1089/acm.2020.0063

[26] Fontana JM, Pan Z, Sazonov ES, McCrory MA, Thomas JG, McGrane KS, et al. Reproducibility of dietary intake measurement from diet Diaries, photographic food Records, and a novel sensor method. Front Nutr. 2020;7:99. 32760735 10.3389/fnut.2020.00099 PMC7372708

[27] Howe M, Leidel A, Krishnan SM, Weber A, Rubenfire M, Jackson EA. Patient-related diet and exercise counseling: do providers’ own lifestyle habits matter? Prev Cardiol. 2010;13(4):180–5. 20860642 10.1111/j.1751-7141.2010.00079.x

[28] Kahan S, Manson JE. Nutrition counseling in clinical practice: how clinicians can do better. JAMA. 2017;318(12):1101–2. 28880975 10.1001/jama.2017.10434

[29] Crowley J, Ball L, Hiddink GJ. Nutrition in medical education: a systematic review. Lancet Planet Health. 2019;3(9):e379–89. 31538623 10.1016/S2542-5196(19)30171-8

[30] Mehrtash F, Manson JE. The 5 a’s approach to promoting nutrition counseling in primary care. J Prim Care Community Health. 2025;16:21501319251338566. 40408085 10.1177/21501319251338566 PMC12102564

