# Supplementary material for: The culinary medicine elective “Prospective Physicians For Fibre” improves fibre intake and nutrition knowledge in German medical students
Source: BMC Med Educ. 2026 Jan 6;26:148. doi: 10.1186/s12909-025-08547-z (PMC12849617; doi:10.1186/s12909-025-08547-z)

# Supplementary Files for:

**The Culinary Medicine Elective “Prospective Physicians for Fibre” Improves Fiber Intake and Nutrition Knowledge in German Medical Students**

# Supplementary Tables

## Supplementary Table 1

Supplementary Table 1 title: Content of the study sessions

| Session 1 | **Main topic: Introduction session**  What is culinary medicine?  What is the purpose of this culinary medicine elective?  Who are the other group members?  What hygiene rules apply in the teaching kitchen?  Miscellaneous |
| --- | --- |
| Session 2 | **Main topic: Fiber intake – Recommendations and assessment**  What is dietary fiber?  Why is dietary fiber beneficial to human health?  What is the difference between soluble and insoluble fibers?  What are short-chain fatty acids?  How can dietary fibers help to lower cholesterol?  How does fiber intake affect bowel health?  How much fiber should humans consume per day?  How can humans increase their fiber intake?  Which foods contain fiber, which not? *(included hands-on exercise)*  What are high-fiber foods? *(included hands-on exercise)*  Which flours have the most fiber? *(included hands-on exercise)*  How does flour processing affect fiber content?  Fiber intake challenges in celiac *(included hands-on exercise)*  Miscellaneous |
| Session 3 | **Main topic: Clinical cases**  Case 1: Colorectal carcinoma and fiber  Case 2: Metabolic syndrome and fiber  Case 3: Chronic constipation and fiber  How could these individuals increase their fiber intake? *(included hands-on exercise)* |
| Session 4 | **Main topic: Legumes and fiber intake**  What are legumes?  Which legumes are available in Germany? *(included hands-on exercise)*  Why are legumes beneficial to human health?  Nutritional properties of legumes? *(included hands-on exercise)*  Legume preparation: pitfalls and caveats *(included hands-on exercise)*  Miscellaneous |

## Supplementary Table 2

Supplementary Table 2 title: Menus prepared in the teaching kitchen

|  | **Menu I (German)** | **Menu I (English)** | **Menu II (German)** | **Menu II (English)** |
| --- | --- | --- | --- | --- |
| Appetizer | Veganer Käse “Cwasare” auf Basis von Blumenkohl und Cashews | Vegan cheese “Cwasare” based on cauliflower and cashews | Linsenwaffeln mit Guacamole auf Erbsenbasis | Lentil waffles with pea-based guacamole |
| Main course | Bowl mit allen Nährstoff-Gruppen:  - Original indisches Linsen-Dhal  - Tofu paniert  - Knusper-Topping  - Rotes Orient-Getreide  - Zitronencrème-Salat  - Gemüse-Nusscrème  - gemischter Salat | Bowl with all nutrient groups:  - Original Indian lentil dhal  - Tofu (breaded)  - Crispy topping  - Red oriental grains  - Lemon cream salad  - Vegetable and nut cream  - Mixed salad | - Würstchen selbst gedreht, gedämpft und gebraten  - Veganer Burgerpatty/Bratling  - Knusprige und cremige Süßkartoffeln  - Majonnaise  - Ketchup ohne Zucker  - Coleslaw  - gemischter Salat | - Sausages self-twisted, steamed and fried  - Vegan burger patty/fried patty  - Crispy and creamy sweet potatoes  - Mayonnaise  - Ketchup without sugar  - Coleslaw  - Mixed salad |
| Dessert | Mousse au Chocolat | Mousse au Chocolat | Heidelbeer-Blitz-Eis | Quick blueberry ice cream |

## Supplementary Table 3

Supplementary Table 3 title: Pre- and post-survey questions

| **F1**: The German Nutrition Society (DGE) currently recommends the following fiber intake for adults aged 25-51 years (in grams per day): |
| --- |
| ⃝ ≥10 g/d  ⃝ ≥15 g/d  ⃝ ≥20 g/d  ⃝ ≥30 g/d  ⃝ ≥40 g/d |

| **F2**: Which of the following foods does not contain relevant amounts of fiber? (Multiple correct answers are possible) |
| --- |
| ⃝ Brown eggs  ⃝ White eggs  ⃝ Atlantic salmon (cooked)  ⃝ Oyster mushrooms (cooked)  ⃝ Eggplant |

| **F3**: Many individuals do not meet the national dietary fiber intake recommendations. In the US, this affects... |
| --- |
| ⃝ less than 10% of the population  ⃝ approximately 30% of the population  ⃝ approximately 50% of the population  ⃝ approximately 75% of the population  ⃝ more than 90% of the population |

| **F4**: Eating dietary fiber offers physiological benefits for human health. Which statement(s) is/are correct? (Multiple correct answers are possible) |
| --- |
| ⃝ Fibers increase the time food stays in the stomach and thus promotes the feeling of satiety  ⃝ Fibers bind water in the intestine and thus increase the stool volume  ⃝ An adequate fiber intake reduces the risk of developing type 2 diabetes mellitus  ⃝ The fermentation of fiber produces anti-inflammatory short-chain fatty acids  ⃝ A regular and sufficient intake of dietary fiber helps to lower LDL cholesterol levels |

| **F5**: Below, four different foods were ranked based on their dietary fiber content in ascending order. Which order is correct? |
| --- |
| ⃝ Broccoli (fresh) --> Avocado (fresh) --> red lentils (raw) --> oatmeal  ⃝ Avocado (fresh) --> Broccoli (fresh) --> --> oatmeal --> red lentils (raw)  ⃝ Broccoli (fresh) --> Avocado (fresh) --> oatmeal --> red lentils (raw)  ⃝ red lentils (raw) --> oatmeal --> Avocado (fresh) --> Broccoli (fresh)  ⃝ The fiber content of the four foods mentioned here is approximately the same |

| **L1**: Which of the following macronutrient profiles best describes a cup of cooked soybeans? |
| --- |
| ⃝ Protein ca. 28 g; fat ca. 15 g; carbohydrate ca. 17 g; fibers ca. 11 g  ⃝ Protein ca. 15 g; fat ca. 11 g; carbohydrate ca. 17 g; fibers ca. 11 g  ⃝ Protein ca. 10 g; fat ca. 30 g; carbohydrate ca. 17 g; fibers ca. 5 g  ⃝ Protein ca. 5 g; fat ca. 15 g; carbohydrate ca. 17 g; fibers ca. 25 g  ⃝ Protein ca. 5 g; fat ca. 5 g; carbohydrate ca. 35 g; fibers ca. 25 g |

| **L2**: According to estimates by the Federal Agricultural Information Centre (Bundes-informationszentrum Landwirtschaft), the current per capita consumption of pulses in Germany is approximately …? |
| --- |
| ⃝ 500 grams  ⃝ 1 kilogram  ⃝ 2 kilograms  ⃝ 5 kilograms  ⃝ 8 kilograms |

| **L3**: Which statement(s) is/are true regarding the nutrient content of legumes? (Multiple correct answers are possible) |
| --- |
| ⃝ They contain virtually no saturated fatty acids  ⃝ The glycemic index of most legumes is between 10 and 40  ⃝ The protein content of beans (per 100 grams) is comparable to that of beef  ⃝ The iron content of beans (per 100 grams) is comparable to that of beef  ⃝ Beans contain less magnesium (per 100 grams) than beef |

| **L4**: Which statement(s) is/are true about legumes? (Multiple correct answers are possible). |
| --- |
| ⃝ Higher legume intake is associated with lower overall mortality  ⃝ Legumes are rich in carbohydrates and should thus be avoided in type 2 diabetes mellitus  ⃝ Legumes contain inositol pentakisphosphate and are therefore unsuitable for pregnant women  ⃝ Legumes have a worse CO2 footprint than common meats (turkey, beef)  ⃝ Kidney beans contain significantly less protein than mung beans and black beans |

| **L5**: Which of the following statements is not correct? |
| --- |
| ⃝ Compared to other beans, black-eyed peas are less likely to cause flatulence and bloating  ⃝ Studies demonstrated that the risk of flatulence after consuming beans is overestimated  ⃝ According to studies, men are more often affected by flatulence after consuming beans than women  ⃝ The fiber content of different types of beans varies  ⃝ After regular consumption of beans, a habituation effect sets in – flatulence and bloating become less frequent and improve after a few weeks |

The correct answers were as follows: Question F1: MC-option 4. Question F2: MC-options 1,2,3. Question F3: MC-option 5. Question F4: MC-options 1,2,3,4,5. Question F5: MC-option 3. Question L1: MC-option 1. Question L2: MC-option 3. Question L3: MC-options 1,2,3,4. Question L4: MC-option 1. Question L3: MC-option 4. MC = Multiple Choice.

## Supplementary Table 4

Supplementary Table 4 title: Free comments: positive (green) and negative feedback (red); unmodified (except for comments regarding personal health) and in German language. A translated version was added for international readers.

| **ID 001** | **German**: Tolles Wahlmodul! Sehr lehrreich, tolle Kochabende!  **English**: Great elective module! Very informative, great cooking evenings! |
| --- | --- |
| **ID 002** | **German:** Sehr informativ, abwechslungsreich, spaßig. Anregung: Mehr auf Patientenkommunikation eingehen.  **English:** Very informative, diversified, and fun. Suggestion: Focus more on patient communication. |
| **ID 003** | **German**: Sehr spannendes Wahlmodul! Die Seminare waren interessant und kurzweilig gestaltet, ich konnte v.a. viel praktisches Wissen für den Alltag (oder zukünftige Patient *innen-Gespräche) mitnehmen. Die Kochabende waren ebenfalls sehr interessant, es gab leckeres Essen, der Koch was sympathisch und es hat Spaß gemacht, gemeinsam mit Kommilitonen zu kochen - das war Uni mal anders!  **English**: A very exciting elective module! The seminars were interesting and engaging, and I gained a lot of practical knowledge for everyday life (or future patient consultations). The cooking evenings were also very interesting; the food was delicious, the chef was friendly, and it was fun to cook together with fellow students – it was a different kind of university experience! |
| **ID 004** | **German**: Ich fand das Modul sehr umfassend und strukturiert (Theorie + Praxis) für die kurze Zeit, die zur Verfügung stand.  **English**: I found the module very comprehensive and well structured (covering theory + practical application) for the short time available. |
| **ID 005** | **German**: Die Seminare waren informativ und interaktiv. Die Kochabende waren überraschend und lehrreich. Wahlmodul zu empfehlen.  **English**: The seminars were informative and interactive. The cooking evenings were surprising and of high educational value. This elective module is to be recommended. |
| **ID 006** | **German**: War von Anfang bis Schluss total begeistert. Die Seminare waren unterhaltsam und lehrreich, so dass Freunde die teilgenommen haben und ich bis heute darüber reden. Gerne auch das Wahlmodul thematisch erweitern, z.B. auf Proteine etc.  **English**: I was absolutely thrilled from the beginning to the end. The seminars were entertaining and informative, so much that my friends who also participated and I still talk about them to this day. Feel free to expand the elective module thematically, e.g., to include protein intake, etc. |
| **ID 007** | **German**: Es wäre auch super Seminare über Proteine, Fette, usw. zu habe. Und vielleicht wäre es auch interessant in den Seminaren über die Anpassung der Ernährungsbedürfnisse an die Physiologie und das Alter zu sprechen.  **English:** It would also be great to have comparable seminars on proteins, fats, etc. And perhaps it would also be interesting to discuss in the seminars how to adapt nutritional needs to physiology and age. |
| **ID 008** | **German**: Gerne noch mehr Fokus auch auf andere Nährstoffe und vorher noch mehr Informationen wie der Ernährungsplan ausgefüllt werden soll.  **English:** It would be interesting to focus more on other nutrients and to have more information beforehand on how to fill out the food diary. |
| **ID 009** | **German**: Mir hat es echt ganz viel Spaß gemacht. Ich habe das Gefühl, viel mitgenommen zu haben. Ich fand es gut, dass Ballaststoffe so intensiv durchgenommen wurden. Die Atmosphäre beim Kochen war super; der Koch, die Location, alles hat wunderbar gepasst. Es war nicht zu viel Druck alles richtig machen zu müssen. Ich fand es auch eine tolle Chance zu sozialisieren und die Gruppe hat sich schnell arrangiert und alle haben sich wohl gefühlt. War ich zur Verbesserung vorschlagen könnte, wäre nur vielleicht, dass der Theorieteil mehr Gewicht bekommt. Ballaststoffe waren ein gutes Thema über das man nicht viel weiß im Allgemeinen, aber es hat meine Neugierde und mein Interesse erst geweckt. Deshalb hätte mich vegane Ernährung vs. Fleisch und Keto etc. auch noch sehr interessiert. Genauso Vitamine, Fette, Kohlenhydrate. Die 1,5h Sessions waren sehr kurzweilig. Eine angenehme Zeitspanne. Man hätte vielleicht noch mehr Theorie reinpacken können. Herr Dr. Storz müsste nicht so entschuldigend sein, dass überhaupt so viel Theorie gemacht wird, sondern sich vielmehr gut dabei fühlen uns Wissen und Informationen zu vermitteln, an die man nicht leicht rankommt. Ich bin normalerweise gar nicht vegan aber die Kochkurse waren vegan was ich gut verstehen kann. Es war auch interessant, in dem Sinne neue Impulse in das eigene Rezeptbuch zu bekommen. Nur wäre da vielleicht noch mehr Wissen dazu sehr spannend gewesen. Meine ganze Kritik soll das Bild nicht trügen: es war das Erste im Studium was so richtig viel Spaß gemacht hat. Vielen Danke, dass Sie das angeboten haben. Ich finde Ernährung fehlt in unsere Ausbildung wirklich sehr. Ich habe mir auch mit allem Wissen eine Mindmap erstellt, die ich mitnehme in Zukunft, damit ich bloß nichts mehr vergesse und an meine Freunde / Familie weitergeben kann. Dankeschön!  **English**: I really enjoyed it. I feel that I learned a lot. I appreciated the in-depth focus on fiber. The atmosphere while cooking was fantastic; the chef, the location, everything was perfect. There wasn't too much pressure to get everything right. I also thought it was a great opportunity to socialize, and the group quickly gelled and everyone felt comfortable. The only thing I could suggest for improvement would perhaps be that the theory section should be given more weight. Dietary fiber was a good topic that one doesn't know much about in general, but it sparked my curiosity and interest. That's why I would have been very interested in vegan nutrition vs. meat and keto diets, etc. Likewise, vitamins, fats, and carbohydrates. The 1.5-hour sessions were very entertaining. A pleasant amount of time. Perhaps even more theory could have been included. Dr. Storz shouldn't be so apologetic about the amount of theory; instead, he should feel good about sharing knowledge and information that isn't readily available. I'm not normally vegan at all, but the cooking classes were vegan, which I can understand. It was also interesting to get new ideas for my own recipe collection. However, even more information on that would have been very exciting. My criticism shouldn't detract from the overall experience: it was the first thing in my studies that I really enjoyed. Thank you so much for offering this. I think nutrition is really lacking in our education. I also created a mind map with all the new information, which I'll take with me in the future so I don't forget anything and can share it with my friends and family. Thank you! |
| **ID 010** | **German**: Die Kochabende waren super. Die Seminare v.a. mit den Lebensmitteln waren spannend. Lernen durch anfassen; würde ich nochmal machen. Gerne auch zu anderen Themen. Vielleicht kann man zudem was Wolfgang Reuter uns erzählt hat oder zu den Seminaren ein Handout verteilen.  **English**: The cooking evenings were fantastic. The seminars, especially those focusing on food, were fascinating. Hands-on learning was the key; I'd definitely do it again. I'd also be happy to see other topics covered. Perhaps you could also distribute handouts about what Wolfgang Reuter told us or about the seminars. |
| **ID 011** | **German**: Kochabende wirklich super, gerne Handouts nach Seminar.  **English**: Cooking evenings were really great, handouts after the seminar would be great. |
| **ID 012** | **German**: Fand das Wahlmodul sehr bereichernd. Sowohl mit Blick auf den Wissenszuwachs durch die Seminare als auch die Kochabende, die viel Spaß gemacht haben. Persönlich habe ich mich sehr gefreut, dass vegane Gerichte gekocht und Background-Wissen vermitteln wurden, da ich selbst vegan lebe. Durch die Studie achte ich beim Einkauf mittlerweile auf den Ballaststoffgehalt der Produkte.  **English**: I found the elective module very enriching. Both in terms of the knowledge gain through the seminars and the cooking evenings, which were a lot of fun. Personally, I was very pleased that vegan dishes were cooked and background information was provided, as I am vegan myself. Thanks to the study, I now pay attention to the fiber content of products when shopping. |
| **ID 013** | **German**: Das Wahlmodul war inhaltlich sehr interessant. Die Seminare auf einem guten Niveau, sodass wir auch ohne viel Vorwissen gut teilnehmen konnten. Das Kochen war sehr lehrreich und hat mir nachhaltig Wissen + Skills auf den Weg gegeben, die ich zuhause bereits angewandt habe. Es hat außerdem mein Interesse am Kochen und Essen gesteigert.  **English**: The elective module was very interesting content-wise. The seminars were of a high standard, so we could participate well even without much prior knowledge. The cooking sessions were of high value and gave me lasting knowledge and skills that I have already applied at home. It also increased my interest in cooking and food. |
| **ID 014:** | **German**: Ich fand die Seminare sehr lehrreich und deutlich weniger langweilig als so manche anderen. Die Kochkurse haben auch Spaß gemacht und ich fand es sehr gut, dass wir vegan gekocht haben. Meine Freunde waren leider in der ersten Gruppe und ich bin nicht so gut in sozialen Situationen. Vielleicht könnte man bei der Einsortierung in die Gruppen kurz fragen, ob man sich gemeinsam mit anderen anmelden möchte.  **English**: I found the seminars very informative and much less boring than some other seminars in my studies. The cooking classes were also fun, and I really appreciated that we cooked vegan food. Unfortunately, my friends were in the first group, and I'm not very good in social situations. Perhaps when assigning participants to groups, you could briefly ask if they would like to register together with others. |
| **ID 015:** | **German**: Es wäre schön gewesen, für die Ernährungsprotokolle etwas mehr Tage zur Auswahl zu haben.  **English**: It would have been nice to have a few more days available to complete the food diaries. |
| **ID 016:** | **German**: Sehr gut & entsprechend organisiert; In dem Format jetzt keine Verbesserungsvorschläge; falls es länger angesetzt werden soll vielleicht immer Theorieseminar mit passendem Praxistag abwechseln.  **English**: Very well organized; no suggestions for improvement in this format; if a longer elective is planned, alternating theory and practice would be nice. |
| **ID 017** | **German**: Ich habe mich auf jedes Seminar und die Kochkurse gefreut und immer etwas Neues mitgenommen. Auch beim Einkaufen hinterfrage ich jetzt, was ich einkaufen muss um möglichst viele Ballaststoffe zu mir zu nehmen und auch inwiefern Marketing „ballaststoffreich“ auf Produkte tatsächlich zutrifft. Schade, dass der Kurs schon vorbei ist.  **English**: I looked forward to every seminar and cooking class and always learned something new. Even when shopping, I now question what I need to buy to maximize my fiber intake and to what extent the marketing claim "high in fiber" actually applies to products. It's a shame the course is already over. |
| **ID 018** | **German**: Ich kauf jetzt tatsächlich anders ein und ernähre mich noch bewusster. Die Seminare haben sehr viel Spaß gemacht und waren interessant. Die Kochkurse waren sehr schön und man konnte viel mitnehmen. Die Ernährung, die man an vier Tagen dokumentiert hat, war natürlich nicht ganz repräsentativ, da das Mensa Essen viel ungesünder ist was man in der Zeit ja nicht essen sollte.  **English**: I'm actually shopping differently now and eating more consciously. The seminars were a lot of fun and very interesting. The cooking classes were great, and I learned a lot. The food I documented over four days wasn't entirely representative, of course, since the cafeteria food is much less healthy, which is something you shouldn't be eating during that time. |
| **ID 019** | **German**: Der Getränkekühlschrank war sehr praktisch, auch sonst war alles supi. Vielleicht hätte der Koch noch ein kleines bisschen mehr auf Ballaststoffe eingehen können, damit ein größerer Bezug zu den Seminaren besteht.  **English**: The drinks in fridge were most welcome, and everything else was great too. Perhaps the chef could have focused a little more on fiber during the cooking sessions. |
| **ID 020** | **German**: Der Kochkurs war sehr lehrreicht; hat auch Spaß gemacht! Vielleicht aber noch ein bisschen mehr darauf eingehen, was jetzt genau im Essen in welcher Menge enthalten ist. Theoretisches Wissen ist besser geworden; habe aber nicht das Gefühl jetzt praktisch besser einschätzen zu können, wie viele Ballaststoffe ich jetzt zu mir nehme.  **English**: The cooking class was very informative and fun! Perhaps it would be good to add a little more detail about the exact ingredients and fiber quantities in the food. My theoretical knowledge has improved, but I don't feel I'm any better equipped now to estimate my fiber intake. |
| **ID 021** | **German**: Sowohl die Seminare, als auch der praktische Teil hat mir sehr gut gefallen!  **English**: I really enjoyed both the seminars and the practical part! |
| **ID 022** |  |
| **ID 023** | **German**: Beim Kochen wäre es gut gewesen, wenn alle jedes Gericht zubereiten würden. Kann aber auch verstehen, dass dies einen zeitlichen Mehraufwand bedeutet. Vielleicht wäre eine zeitliche + thematische Verknüpfung der Seminare und Rezepte möglich. Theorie-Praxis-Theorie-Praxis und nicht erst den ganzen Theorie-Block.  **English**: It would have been good if everyone had prepared each dish during the cooking session. However, I understand that this would take more time. Perhaps a chronological and thematic linking of the seminars and recipes would be possible. Alternate theory-practice-theory-practice, rather than a whole block of theory first. |
| **ID 024** | **German**: Fand das Wahlmodul PPFF richtig gut; es war eine Bereicherung teilnehmen zu dürfen. Aufgrund von Zeitmangel konnte der theoretische Input nicht ausführlicher sein. Da mich persönlich das Thema interessiert, hätte ich mich gefreut, mehr Seminare zum Thema besuchen zu dürfen. Die Kochkurse mit dem Koch waren ein echtes Highlight. Mir hat die entspannte Atmosphäre, die Freundlichkeit sowohl vom PPFF-Team als auch vom Koch sehr gut gefallen. Ich habe viel mitgenommen und würde sogar so weit gehen und sagen, dass ich mir mehr Gedanken bei meiner Ernährung mache. Vielen Dank!  **English**: I really enjoyed the PPFF elective module; it was a valuable experience to participate. Due to time constraints, the theoretical input couldn't be more comprehensive. Since I'm personally interested in the topic, I would have liked to attend more seminars on it. The cooking classes with the chef were a real highlight. I appreciated the relaxed atmosphere and the friendliness of both the PPFF team and the chef. I learned a lot and would even go so far as to say that I'm now giving more thought to my diet. Thank you! |
| **ID 025** | **German**: Seminar: gute Länge, interessant, Gruppenarbeit gut. Bereitstellen der Folien wäre hilfreich. Kochkurs: gute Gruppengröße, schöne Location. Vielleicht mehr Partizipation bei der Auswahl der Gerichte. Reihenfolge: Seminare und Kochkurse im Wechsel?  **English**: Seminar: good length, interesting, good group work. Providing the slides would be helpful. Cooking class: good group size, nice location. Perhaps students should be involved more in the selection of dishes. You may alternate the sequence of the seminars and cooking classes. |
| **ID 026** | **German**: Schöne und angenehme Möglichkeit sich tiefer mit dem Thema auseinander zu setzen. Kochen in der Gruppe macht Spaß und man lernt voneinander / nimmt wirklich was mit. Ich hoffe es wird als Wahlfach kommen!  **English**: A wonderful and enjoyable way to delve deeper into the subject. Cooking in a group is fun, and you learn from each other. I hope it will be offered as an elective! |
| **ID 027** | **German**: Die Studie hat nicht nur die eigenen Kenntnisse über ballaststoffreiche Ernährung erweitert, sondern auch die Bedeutung von Ernährung als Bestandteil der Medizin verdeutlicht. Zusätzlich zur Teilnahme konnte das erlernte Wissen in Form von Kochabenden angewandt und vertieft werden. Demnach ist das damit verbundene Wahlfach in meinen Augen eine Bereicherung für den Studenten und seine spätere Tätigkeit als Arzt.  **English**: The study not only expanded my knowledge of high-fiber diets but also highlighted the importance of nutrition as an integral part of medicine. In addition to participation, the acquired knowledge could be applied and reinforced through cooking evenings. Therefore, in my opinion, this elective course is of high value for every student and their future career as a physician. |
| **ID 028** | **German**: Es hat sehr viel Spaß gemacht und ich haben viel für mich selbst gelernt. Insbesondere zu Ballaststoffen und wie man sie im Alltag integrieren kann. Es war super, dass wir uns mit den Lebensmitteln auseinandersetzen mussten. Durch die Gruppenarbeit und Besprechungen ist dann sehr viel hängen geblieben. Muss unbedingt Wahlfach werden.  **English**: It was a lot of fun and I learned a great deal, especially about fiber and how to incorporate it into everyday life. It was fantastic that we had to engage with food in a hands-on way. The group work and discussions really helped me retain a lot of information. It absolutely has to become an elective. |
| **ID 029** | **German**: Hätte gerne noch mehr über andere Ernährungsbestandteile gelernt. Sehr cooles Wahlfach!  **English**: I would have liked to learn more about other nutrients. A really cool elective! |
| **ID 030** | **German**: Super wichtiges Thema! Daher gut, Mediziner über praktische Anwendung und Theorie aufzuklären und Ihnen Spaß am Thema zu vermitteln. Seminare mit Fallbeispielen und Gruppenaufgaben gut gestaltet. Kochkurse lehrreich und witzig. Gerne auch Theorie + Praxis abwechselnd. Danke fürs Engagement!  **English**: A very important topic! It's great to educate medical professionals about practical applications and theory, and to make the subject enjoyable for them. Seminars with case studies and group exercises are well-designed. Cooking classes are both informative and fun. Alternating theory and practice would also be welcome. Thank you for your dedication! |
| **ID 031** | **German**: Die Kochabende waren super, sowohl in Bezug auf die Information als auch auf den Spaß! Die Seminare waren sehr interessant, noch mehr Fokus auf konkrete Empfehlungen für einen selbst bzw. gegenüber Patienten wären hilfreich. Also z.B. in Form von Tipps für Gerichte, Nährwertumrechnung, etc.  **English**: The cooking evenings were fantastic, both in terms of information and fun! The seminars were very interesting, but a greater focus on concrete recommendations for oneself or for patients would be helpful. For example, tips for dishes, nutritional information, etc. |
| **ID 032** | **German**: Ich fand die Seminare sehr informativ und habe das Gefühl, dass sich Theorie und Praxis gut ergänzen. Ich kann den Ballaststoffgehalt von Nahrungsmitteln deutlich besser einschätzen und mir in nun deutlich bewusster, dass eine ausreichende Ballaststoff-Zufuhr wichtig ist.  **English**: I found the seminars very informative and felt that theory and practice complement each other well. I can now assess the fiber content of foods much better and am much more aware of the importance of an adequate fiber intake. |
| **ID 033** | **German**: Vielleicht nicht ausschließlich vegane Kochabende, sondern auch mal vegetarisch ☺ Ansonsten sehr cooles Wahlfach!  **English**: Perhaps not exclusively vegan cooking evenings, but also vegetarian ones ☺ Otherwise, a very cool elective course! |
| **ID 034** | **German**: Modul war super interessant. Habe mehr gelernt als in den meisten Uni-Veranstaltungen. Gerne die Rezepte / Gerichte bei den Kochabenden etwas leichter & zeitsparender machen, sodass es zuhause reproduzierbarer ist.  **English**: The module was super interesting. I learned more than in most university courses. It would be great if the recipes/dishes for the cooking evenings could be made a bit easier and more time-saving, so they're easier to recreate at home. |
| **ID 035** | **German**: Insgesamt hat es mir sehr gut gefallen. Man hat super viel für sich und seine Ernährung gelernt und ich fühle mich nach der Studie schon etwas sicherer bei der Beratung von Patienten. In einem Wahlfach wäre es cool tatsächlich Materialien (bspw. Für die Seminare) wie Übersichten mit an die Hand zu bekommen.  **English**: Overall, I really enjoyed it. I learned a great deal about myself and my diet, and I already feel more confident advising patients after the study. It would be great if, in an elective course, we actually received handouts (e.g., for the seminars), such as overviews and summaries. |
| **ID 036** | **German**: Super! Vielleicht die Seminare länger machen, damit mehr Raum für Diskussionen da [ist].  **English**: Great! Perhaps make the seminars longer so that there is more time for discussion. |
| **ID 037** |  |
| **ID 038** | **German**: Die Lehrküche war wirklich toll! Ich habe viel gelernt und es hat sehr Spaß gemacht. Das würde ich jederzeit wiederholen.  **English**: The teaching kitchen was really great! I learned a lot and it was a lot of fun. I would do it again anytime. |
| **ID 039** | **German**: Gute[r] [Erkenntnisgewinn] [über] Ballaststoffe, wäre schön das Thema zu erweitern [um] Proteine oder andere Vitamine.  **English**: Good insights about dietary fiber, it would be nice to expand the topic to include proteins or other vitamins. |
| **ID 040** | **German**: Ich fand es sehr interessant. Als Anregung hätte ich nur, dass es schön wäre eine Art Handout o. Folien zu erhalten.  **English**: I found the elective very interesting. My only suggestion would be that it would be nice to receive some kind of handout or slides. |
| **ID 041** | **German**: Seminare waren super gemacht und sehr informativ. Kochabende haben sehr viel Spaß gemacht. Man hat selbst viel mitgenommen und neue Leute kennengelernt. Gerne auch auf andere Nahrungsbestandteile eingehen. Bei den Kochabenden auch Snacks / Vesper vorstellen.  **English**: The seminars were excellent and very informative. The cooking evenings were a lot of fun. One learned a lot and met new people. It would be good to also cover other food components. Snacks/light meals could also be presented at the cooking evenings. |
| **ID 042:** | **German**: Gerne in den Seminaren über Protein berichten, da Sarkopenie auch ein wichtiges Thema ist. Andere Themenvorschläge: Nahrungsergänzungsmittel. Die Seminare waren sehr lehrreich / nicht langweilig. Die Kochkurse waren mega gut und nötig; es wäre gut wenn es weiter als Wahlmodul angeboten wird.  **English**: I'd be happy to cover protein in the seminars, as sarcopenia is also an important topic. Other topic suggestions: dietary supplements. The seminars were very informative and not at all boring. The cooking classes were fantastic and much needed; it would be great if they continued to be offered as an elective. |
| **ID 043** | **German**: Hat viel Spaß gemacht & habe viel gelernt. Gerne noch mehr Seminare / Infos zu anderen Themen, z.B. Proteine. Verstehe aber, dass es zu umfangreich für die zur Verfügung stehende Zeit gewesen wäre. Danke!  **English**: It was a lot of fun and I learned a lot. I'd love to see more seminars/information on other topics, e.g., proteins. However, I understand that it would have been too extensive for the time available. Thank you! |
| **ID 044** | **German**: Im Wahlfach könnte man eventuell mit mehr Zeit noch ein breiteres Themenspektrum behandeln.  **English**: With more time it might be possible to cover an even broader range of topics in the elective course. |
| **ID 045** | **German**: Stimmung, Seminare, Kochkurs und Team sehr gut. Vorschlag: vielleicht die Seminar- Folieren veröffentlichen oder Unterlagen freigeben. Hat sehr viel Spaß gemacht; konnte viel lernen.  **English**: The atmosphere, seminars, cooking class, and team were all excellent. Suggestion: perhaps publish the seminar slides or make the materials available. It was a lot of fun; I learned a great deal. |
| **ID 046** | **German**: Hat sehr viel Spaß Gemacht und habe viel gelernt! ☺ Gerne schon ab 17 Uhr zum Kochen treffen, damit man früher essen kann.  **English**: It was a lot of fun and I learned a lot! ☺ It would be great to schedule the cooking at 5 pm |
| **ID 047** | **German**: Sehr lehrreiches Modul; sowohl mit praktischen als auch theoretischen Einheiten. Fand ich sehr komplett und spannend. Wenn man noch ein Handout mitnehmen könnte wäre super.  **English**: A very informative module, with both practical and theoretical components. I found it very comprehensive and engaging. It would be great if a handout was available. |

# Supplementary Figures

## Supplementary Figure 1

Supplementary Figure 1 title: Participant inclusion flowchart


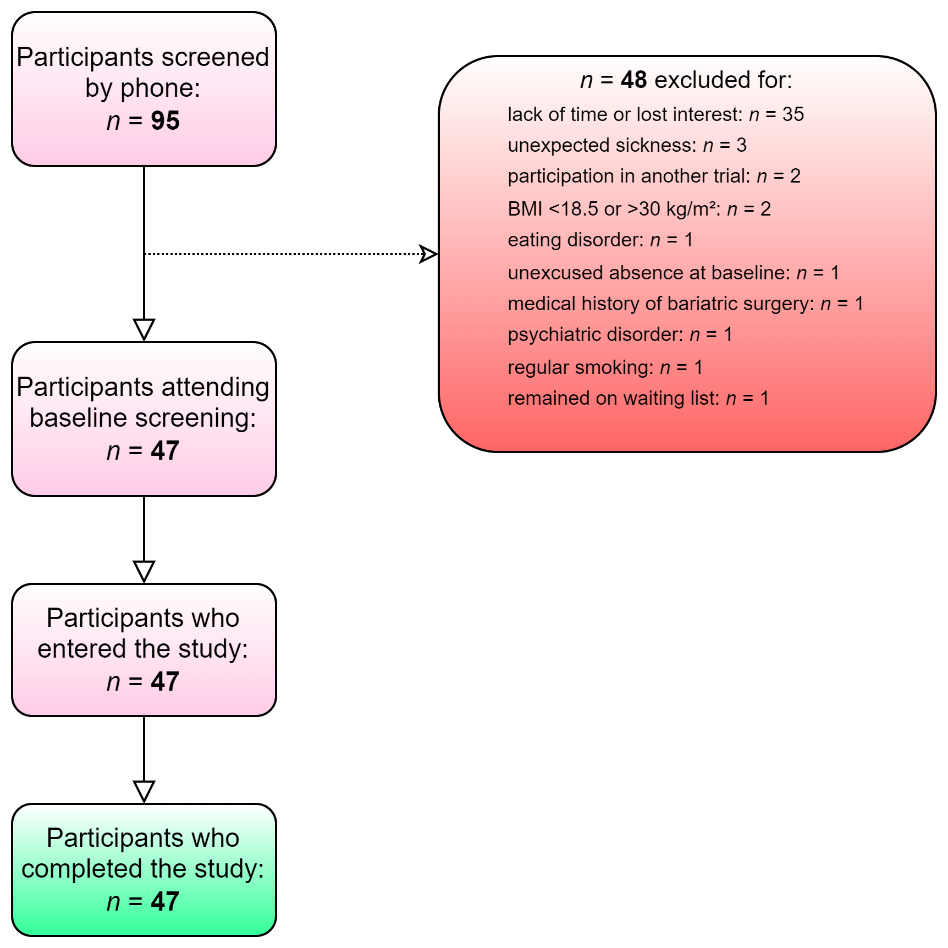


## Supplementary Figure 2

Supplementary Figure 2 title: Bar charts displaying the results of the course grading by students (panel A), and for the Likert scale-based questions on whether participants would re-take the elective without receiving a remuneration (panel B) and whether participants would recommend the elective to their peers (panel C).


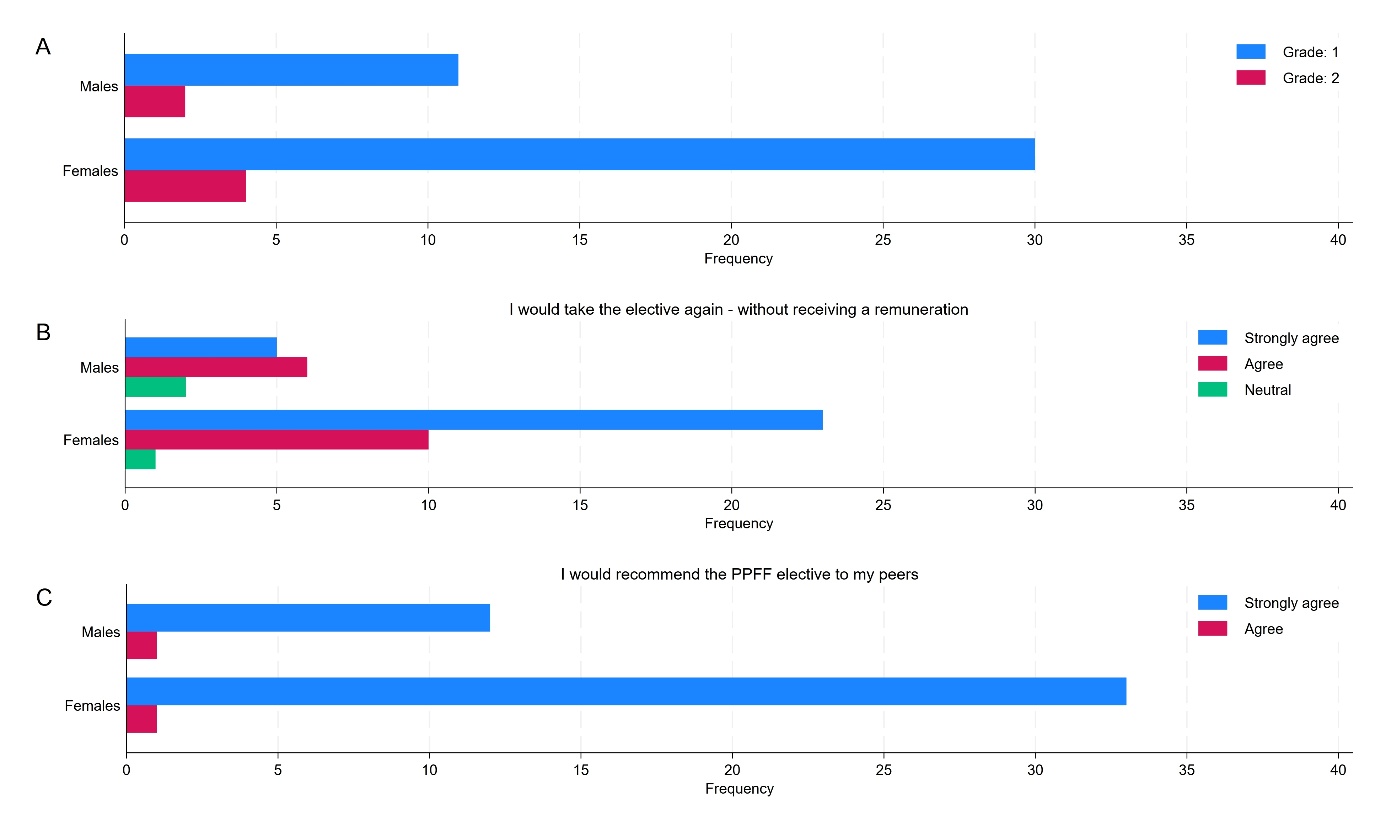


## Supplementary Figure 3

Supplementary Figure 3 title: Bar charts displaying pre-post comparisons for the 5-point Likert scale-based questions in the PPFF survey (questions 1-5)


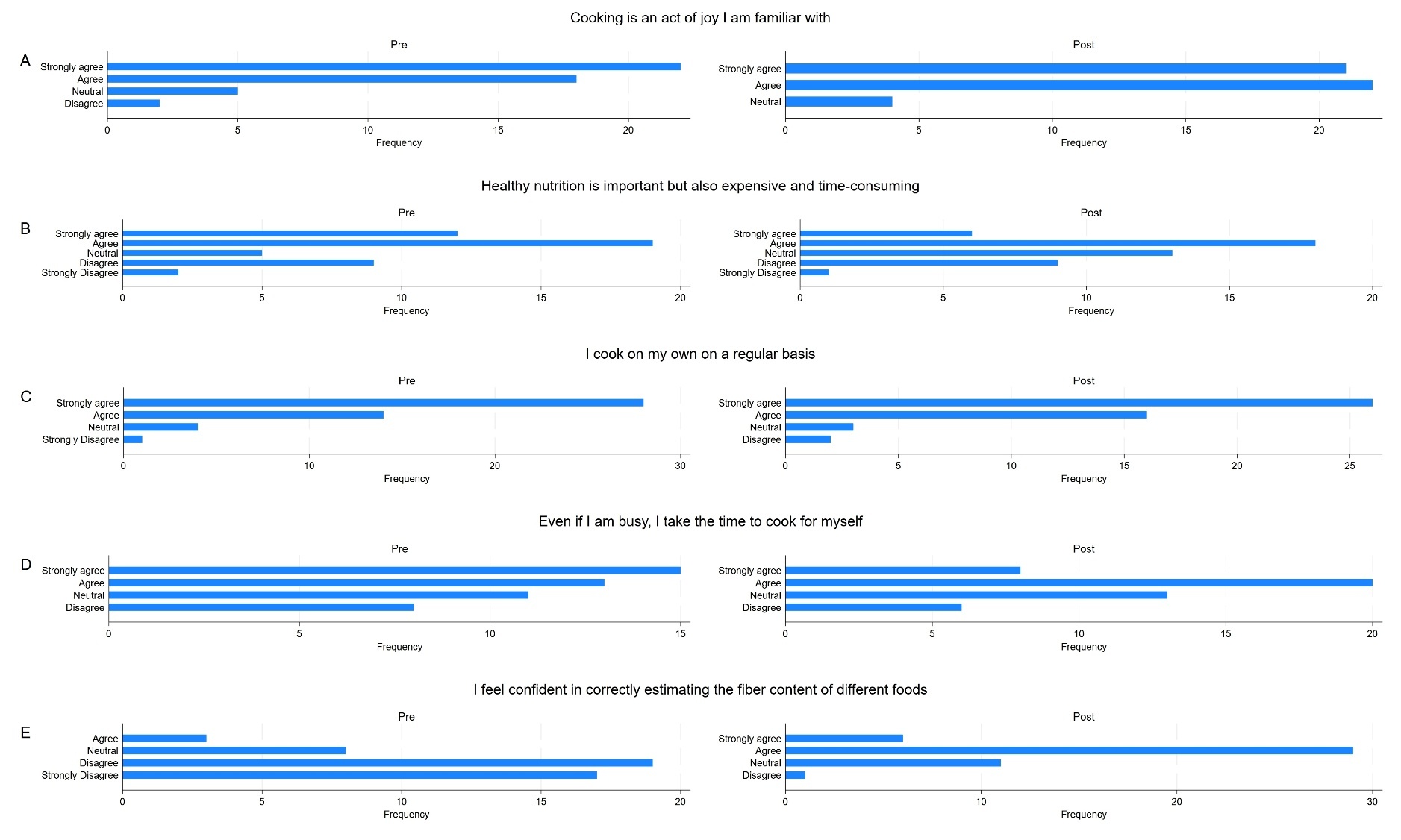


## Supplementary Figure 4

Supplementary Figure 4 title: Bar charts displaying pre-post comparisons for the 5-point Likert scale-based questions in the PPFF survey (questions 6-10)


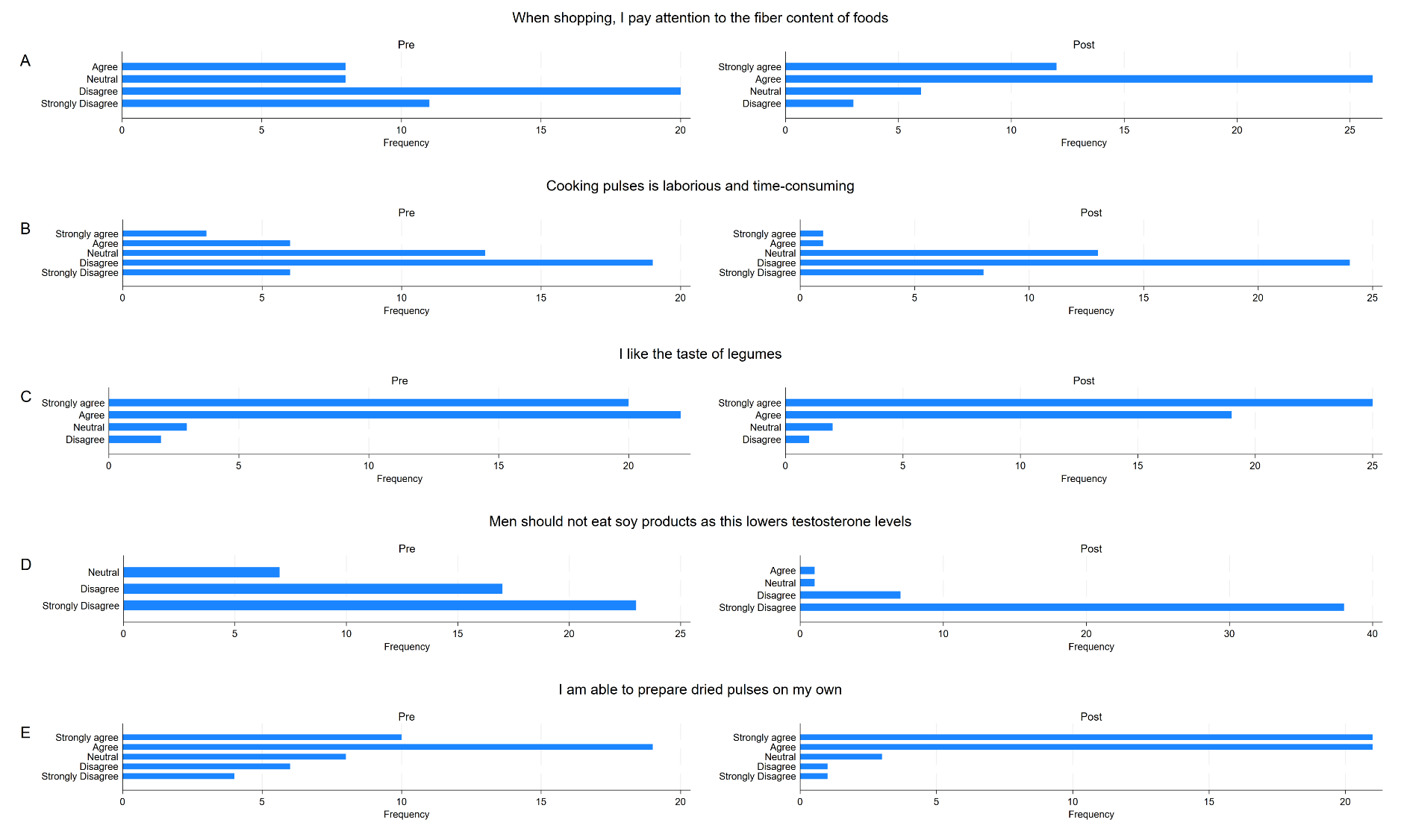


## Supplementary Figure 5

Supplementary Figure 5 title: Bar charts displaying pre-post comparisons for the 5-point Likert scale-based questions in the PPFF survey (questions 11-15)


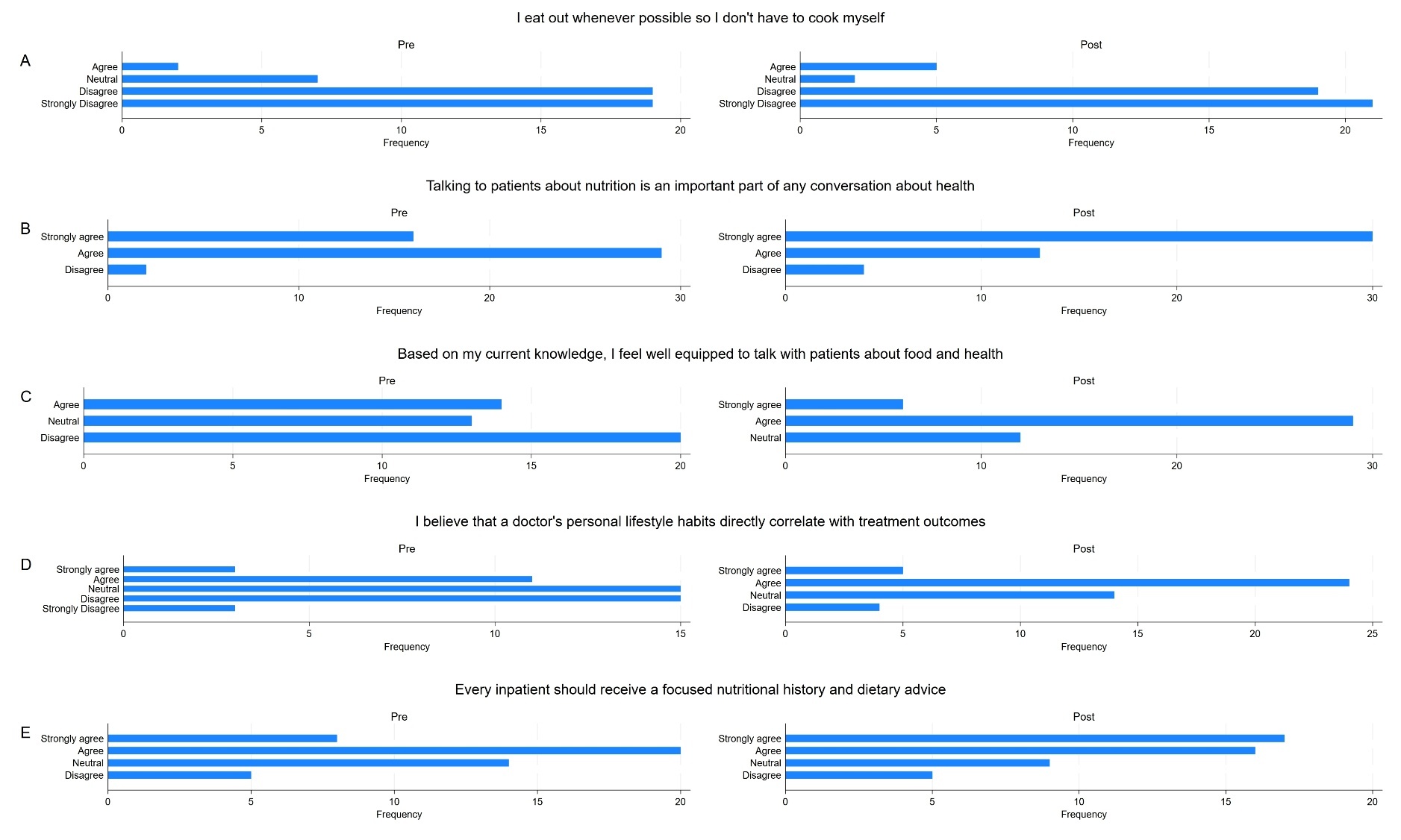


## Supplementary Figure 6

Supplementary Figure 6 title: Bar charts displaying pre-post comparisons for the 5-point Likert scale-based questions in the PPFF survey (questions 16-20)


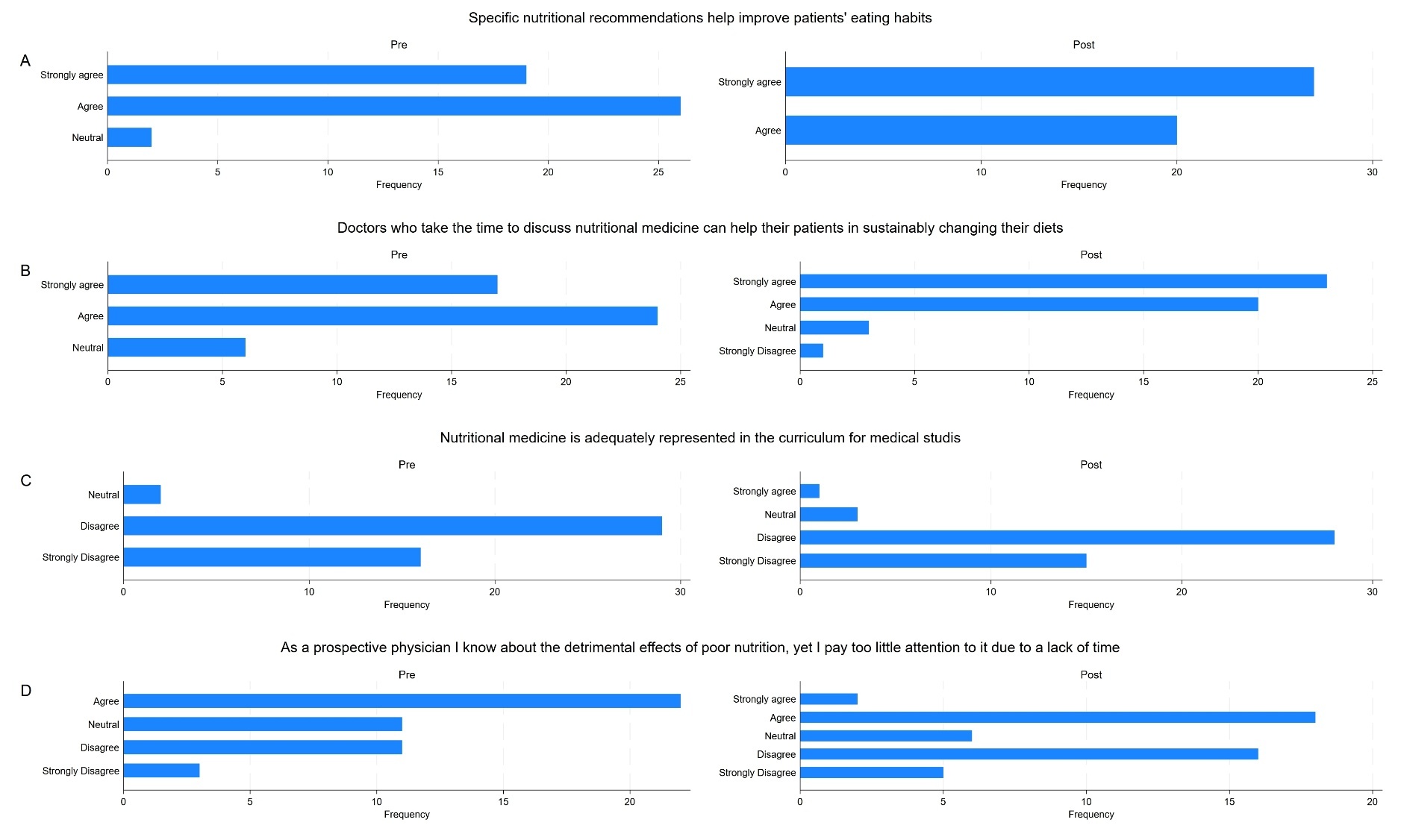


## Supplementary Figure 7

Supplementary Figure 7 title: Strip plots displays students self-rated confidence in their own cooking and nutrition counseling skills in a pre-post comparison


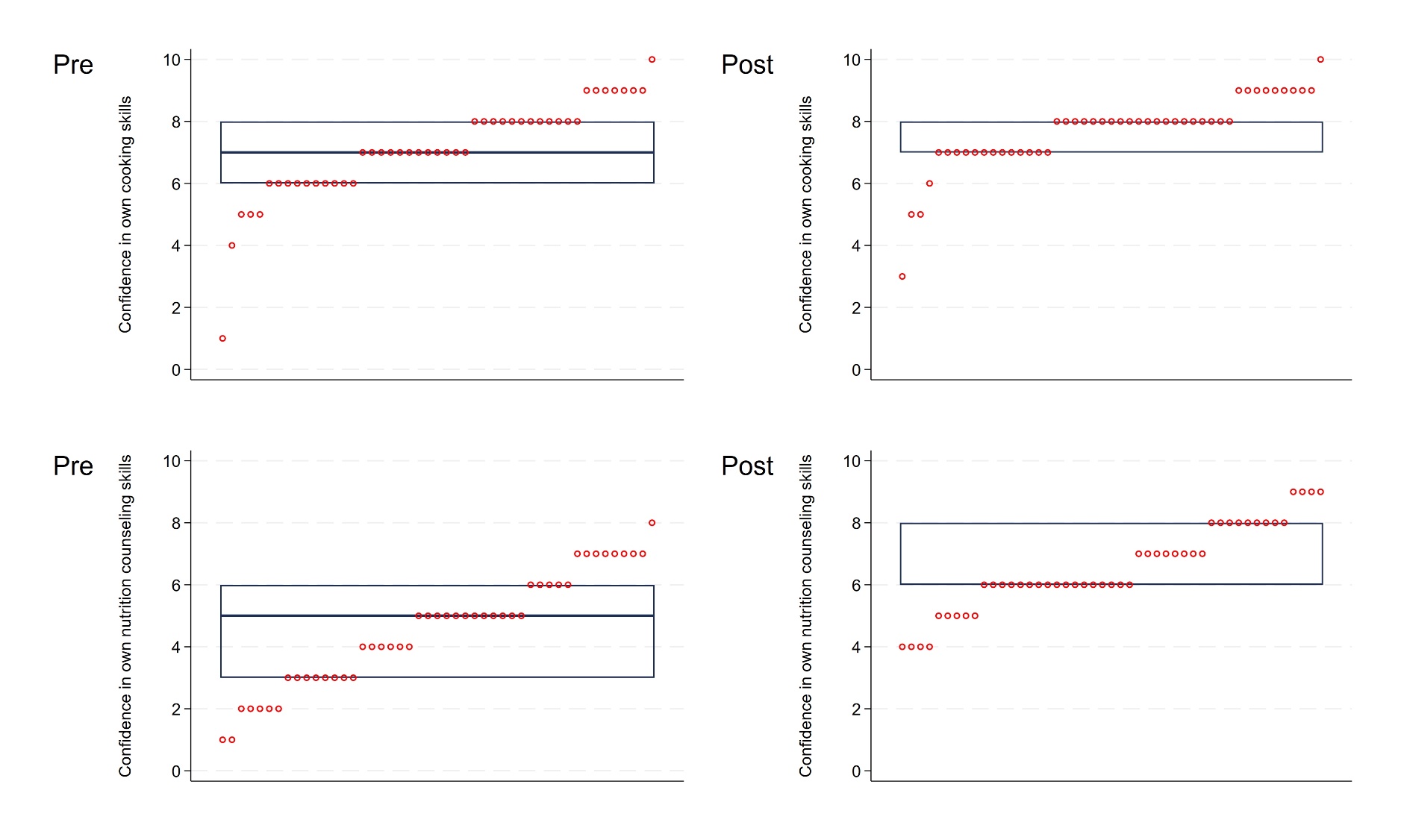

Supplement: Supplementary file 1 — Supplementary Material 1. [file 12909_2025_8547_MOESM1_ESM.docx]
